# The Physiological Response of Two Green Calcifying Algae from the Great Barrier Reef towards High Dissolved Inorganic and Organic Carbon (DIC and DOC) Availability

**DOI:** 10.1371/journal.pone.0133596

**Published:** 2015-08-12

**Authors:** Friedrich Wilhelm Meyer, Nikolas Vogel, Mirta Teichberg, Sven Uthicke, Christian Wild

**Affiliations:** 1 Department of Ecology, Leibniz Center for Tropical Marine Ecology (ZMT), Bremen, Germany; 2 Australian Institute of Marine Science, Townsville, Queensland, Australia; 3 Faculty of Biology and Chemistry, University of Bremen, Germany; Griffith University, AUSTRALIA

## Abstract

Increasing dissolved inorganic carbon (DIC) concentrations associated with ocean acidification can affect marine calcifiers, but local factors, such as high dissolved organic carbon (DOC) concentrations through sewage and algal blooms, may interact with this global factor. For calcifying green algae of the genus *Halimeda*, a key tropical carbonate producer that often occurs in coral reefs, no studies on these interactions have been reported. These data are however urgently needed to understand future carbonate production. Thus, we investigated the independent and combined effects of DIC (*p*CO_2_ 402 μatm/ pH_tot_ 8.0 and 996 μatm/ pH_tot_ 7.7) and DOC (added as glucose in 0 and 294 μmol L^-1^) on growth, calcification and photosynthesis of *H*. *macroloba* and *H*. *opuntia* from the Great Barrier Reef in an incubation experiment over 16 days. High DIC concentrations significantly reduced dark calcification of *H*. *opuntia* by 130 % and led to net dissolution, but did not affect *H*. *macroloba*. High DOC concentrations significantly reduced daily oxygen production of *H*. *opuntia* and *H*. *macroloba* by 78 % and 43 %, respectively, and significantly reduced dark calcification of *H*. *opuntia* by 70%. Combined high DIC and DOC did not show any interactive effects for both algae, but revealed additive effects for *H*. *opuntia* where the combination of both factors reduced dark calcification by 162 % compared to controls. Such species-specific differences in treatment responses indicate *H*. *opuntia* is more susceptible to a combination of high DIC and DOC than *H*. *macroloba*. From an ecological perspective, results further suggest a reduction of primary production for *Halimeda*-dominated benthic reef communities under high DOC concentrations and additional decreases of carbonate accretion under elevated DIC concentrations, where *H*. *opuntia* dominates the benthic community. This may reduce biogenic carbonate sedimentation rates and hence the buffering capacity against further ocean acidification.

## Introduction

Marine calcifiers are facing both global and local threats due to human induced environmental changes. On a global scale, increased emissions from fossil fuel combustion lead to an increased carbon dioxide (CO_2_) concentration in the atmosphere [1]. The dissolution of an increased amount of CO_2_ in the world’s ocean leads to an elevated bicarbonate concentration and a lowered pH of the oceans, causing ocean acidification (OA) and reducing the saturation state (Ω) of carbonates in seawater. Depending on different scenarios, the atmospheric concentration of CO_2_ is predicted to rise from today’s level of app. 396 μatm [2] to between 850 and 1370 μatm by the year 2100 [3]. This increase in CO_2_ or dissolved inorganic carbon (DIC) in the water affects marine life [4–6], and special focus has been put on the effects on marine calcifiers [7–11] which may exhibit a resulting loss in calcification and a weakened carbonate structure [5,12].

Calcifying green algae of the genus *Halimeda* play a major role in sediment formation and provide habitat for many species [13–16]. Because of their high abundance and fast growth[17], *Halimeda opuntia* (Linnaeus) J.V. Lamouroux, 1816 is one of the most prominent species of its genus, also in the Great Barrier Reef (GBR). *H*. *opuntia* represents the “sprawler” type, spreading out and growing attached to rock, sand or soft substrate; whereas another species of this genus, *Halimeda macroloba*, Decaisne, 1841, is a typical sand dweller, usual found in lagoon-like environments. Hence, recent studies investigated the effect of OA on the calcification of different species of *Halimeda* and found that calcification was reduced in terms of needle size of the calcium carbonate deposited or reduced inorganic carbon content, while photosynthetic activity was stable or increased by OA compared to controls [7,18–22].

In addition to the global threat OA, marine life is often also facing disturbances on a local scale, such as increased riverine runoff high in inorganic and organic nutrients and sediments. Reefs at inshore locations of the GBR are more exposed to these threats and already undergo changes leading to a shift from coral-dominated to more macro-algae-dominated reefs [23–25]. An often underrepresented chemical parameter in monitoring of water quality is dissolved organic carbon (DOC) which can fuel bacterial and pathogen growth as observed under the addition of labile sugars [26,27] as well as natural DOC sources [28] and thereby has severe effects on corals [29,30], leading to bleaching, disease spread and eventually mortality [29,30]. Average DOC concentrations in the GBR are 66 μmol L^-1^ [31] and can increase in flood plume to over 200 μmol L^-1^ [31] or even 583 μmol L^-1^ (7 mg L^-1^) [32] or higher in other reef settings [29]. Elevated DOC concentrations, containing highly bioavailable molecules, such as sugars and amino acids, are a result of mostly two processes: increased sewage input connected with river runoff of agricultural land [32–34] and increased release of DOC into the surrounding water by primary producers, mainly benthic and/or planktonic algae [27,35–39]. As the latter is associated with dominance shifts from hard corals to algae during phase shifts, their contribution to the available DOC pool becomes more important [27,38,40]. Climate change and increased storm frequencies with more pronounced seasons and higher precipitation [41,42] may also cause higher discharge of rivers, very likely resulting in increased DOC inputs into inshore waters of the GBR that have been found to correlate with high water discharge rates of rivers to the GBR [31,43]. Higher river inputs of DOC into marine waters can also carry less labile, refractory material which is not metabolized as fast by bacteria like as labile sugars [28,44], but can be co-metabolized under the presence of additional labile organic carbon [45].

Keeping in mind the known severe effects of DOC on corals, such as increased bacterial growth and disease spread, bleaching and mortality [26,29,40,46], it is surprising that no comparable studies have investigated the effects of elevated DOC on *Halimeda* or other calcifying algae. DOC as a local factor may occur simultaneously with other factors, such as DIC, which occurs on a global scale. Such multi-factor settings may show additive or even synergistic effects of the individual factors. In addition, antagonistic interaction may occur, where one factor reduces the effect of the other, resulting in a decreased organism response. To understand and predict the consequences of multiple factors on key reef species functioning, it is essential to conduct combined manipulation experiments.

Hence, in this study we investigated the independent and combined effect of high DIC and DOC concentrations on the physiology of two key calcifying green algae, *H*. *opuntia* and *H*. *macroloba* during a 16 day laboratory experiment.

Under elevated DIC concentration, we expect the calcification of both green algae to decrease and alter the inorganic carbon content of the algae. In addition, photosynthesis and connected photosystem parameters such as F_v_/F_m_ and chlorophyll a content will likely decrease. Nutrient uptake may increase in order to compensate for decreased photosynthetic efficiency. Under elevated DOC concentrations, we hypothesise bacterial growth and connected bacterial respiration to increase and to negatively affect algal fitness, leading to reduced photosynthesis and chlorophyll a contents and ultimately to a reduction of calcification rates. Due to the growing bacterial numbers, we predict nutrient cycling to be enhanced. Hence, uptake rates of organic and inorganic nutrients may increase. Under the combination of both factors, we anticipate additive and synergistic effects leading to further reduced primary production and calcification. In response to both treatments, the physiological parameters measured may show correlation and give indication to what extent the physiology of the whole alga is altered.

## Material and Methods

### Specimen collection and preparation

Individual thalli of both species *H*. *opuntia* (50 to 100 segments per thallus) and *H*. *macroloba* (10–30 segments per thallus) were collected from reefs at Orpheus Island (S 18° 36.737’, E 146° 29.110’) under a GBMPA sampling permit G12/35236.1. Sand dwelling *H*. *macroloba* were potted into 80 mL plastic containers with ordinary beach sand. Both species were kept in 18 L experimental flow-through aquaria under controlled conditions (LED light, Aqua Illumination, 150 μmol photons m^-2^ s^-1^ at 12h/12h light-dark cycle, temperature 25°C, salinity 34.3) for two weeks prior to the start of the experimental treatments.

### Experimental setup

Prior to the experiment, two thalli of each alga species were randomly re-allocated to each of the experimental treatments, as described in detail below. The experiment was conducted over 16 days between 24 July and 9 August 2012 at the Australian Institute of Marine Science (AIMS). Three replicate open glass tanks of 18 L volume for the two treatments with different treatment levels were placed in alternating order (a total of 12 tanks, 3 controls, 3 high DIC, 3 high DOC and 3 high DIC and DOC). The treatments were *p*CO_2_ in ambient and high availability (403 μatm and 996 μatm, respectively) and DOC in ambient and high availability (83 ± 10 and 294 ± 506 μmol L^-1^ with DOC added as glucose (D-glucose, Sigma Aldrich, purity > 99.5%). The target *p*CO_2_ was 1000 μatm and was reached by pH manipulation via pure CO_2_ gas injection by a potentiometric pH sensors controlled pH stat system (Aqua Medic, Germany) as described in Vogel and Uthicke [47]. The pH sensors were calibrated daily using Mettler Toledo NIST-/DIN- pH buffers. The *p*CO_2_ levels corresponded to concentrations likely to be reached under the ‘representative concentration pathways’ (RCP) 6.0 to RCP 8.5 for the year 2100 [1,48,49]. DOC treatment concentrations were chosen in relation to previously described concentrations for coral reefs [29,40] and treatments applied in other studies [26,29,30]. Glucose, a sugar produced by algae and corals [38] was chosen as DOC enrichment as it is easy obtainable, shows high bacterial availability and also eases replication. Other sources of DOC such as the collection of algal or coral exudates were not feasible for the extent of this study and quantification, as well as qualification of the DOC used for the treatment would be challenging. The DOC treatment was achieved by additions of 1170 μmol L^-1^ DOC (Glucose, D-Glucose, Sigma Aldrich) twice daily at 8:00 and 20:00. Subsequent dilution resulted in average concentrations of 294 μmol L^-1^ over 12 h. To determine the resulting DOC concentrations due to dilution in the treatments, we measured the DOC concentrations in duplicates from 8:00 after the glucose addition every hour for three hours and then every other hour for eight hours until 18:00. Flow-through rates for the tanks were adjusted to 150 mL min^-1^ using freshly filtered (0.5 μm) natural seawater to avoid accumulation of particles that had not been previously captured by the settlement tanks. To provide additional water movement, aquaria pumps (AquaWorld, Australia, 250 L h^-1^) were placed in each specimen tank. To determine alkalinity (A_T_), a 50 ml water sample of each tank was drawn at the beginning, middle and end of the experiment. It was measured by gran-point titration with a Metrohm 855 autosampler (Metrohm, Switzerland) using 0.5 M HCl [50] and was corrected to certified reference material (CRM Batch 106, A. Dickson, Scripps Oceanographic Institute). Carbonate system parameters were calculated with CO2calc software [51] from A_T_ in combination with pH readings obtained from a multiprobe (WTW 3430, Germany) calibrated with Mettler Toledo NIST-/DIN- pH buffers.

### Measurement of water column Biological Oxygen Demand (BOD)

To assess effects of elevated organic or inorganic carbon availability on microbial respiration rates, BOD of the treatment water was measured at the end of the experiment for each treatment tank (n = 3). For this purpose, 200 mL of unfiltered seawater were incubated for 24 h in the dark under temperature conditions of the treatments in borosilicate bottles. The O_2_ concentration (mg L^-1^ and % saturation) as well as salinity and temperature were recorded before and after the incubation, and O_2_ consumption rates were calculated from these two values and related to water volume and time to mg O_2_ L^-1^ h^-1^.

### Quantification of algae surface area

In order to relate the measured rates of calcification, photosynthesis and nutrient fluxes to individual surface area of the thalli, the surface area was determined using images of the flattened algae and Image J software. Surface area varied for *H*. *opuntia from* 5 to 35 cm² and for *H*. *macroloba* from 4 to 30 cm².

### Quantification of Light-/ dark calcification and nutrient fluxes

After 16 days under experimental conditions, two algal thalli from each replicate tank were transferred into individual acid-washed nalgene chambers (200 mL) and incubated for 60 min in light and 60 min in darkness. Water for the incubations was taken from the treatments (filtered, 0.5 μm) of the corresponding organisms. Therefore, pH of the incubations corresponded to treatment conditions. DOC in the form of Glucose was added in the concentration of 940 μmol L^-1^ prior to each incubation to assure similar concentrations. The background concentration of DOC was checked before the incubation and was 83 ± 10 μmol L^-1^. Incubations were performed in 12 parallel incubations including two blanks per treatment. To meet light conditions similar to the treatment tanks, individual white LEDs (4000 K, Megaman, Germany) were installed above each incubation chamber and adjusted individually, using a quantum sensor (Apogee, USA). During the incubation, chambers were placed into a temperature controlled water bath at 25°C, equal to the temperature during the 16 day incubation. Water movement during the incubation time was maintained using glass-coated magnetic stir bars driven by magnetic stir plates.

Light- and dark calcification rates were determined by the change of A_T_ during the incubation related to blanks using the alkalinity anomaly technique [52]. To achieve this, a sample of 50 mL incubation water was titrated as described above for the carbonate system parameters. A_T_ was calculated by non-linear regression fitting between pH 3.5 and pH 3.0. Calcium carbonate precipitation or dissolution expressed in μM C h^-1^ was calculated by half molar of the difference between the post incubation and the blank seawater A_T_ readings, volume of chamber, time of incubation and organism surface area [53]. For the calculation of daily calcification rates, the data were extrapolated for a 12 h light and 12 h dark cycle using light calcification and dark calcification data obtained from one h incubations.

Nutrient fluxes in the chambers were determined by analysing sub-samples of seawater from light and dark incubations for dissolved inorganic nutrients (DIN) as ammonium (NH_4_^+^), phosphate (PO^3-^) and nitrite (NO_2_^-^) + nitrate (NO_3_^-^) as NO_x_ and total organic carbon (TOC as Non Particulate Organic Carbon) directly subsequent to the experimental runs. Samples for DIN were filtered using 0.45 μm syringe filters and kept frozen at -20°C until measurement by segmented flow analysis (Seal Analytical, USA). Samples for DOC were filtered through 0.45 μm, acidified with 150 μL fuming HCl and frozen at -20°C until analysis on a Shimadzu TOC-5000A (Shimadzu, USA). Nutrient fluxes were calculated and corrected for the fluxes of the blank incubations, related to organism surface area and expressed as μmol cm² h^-1^.

### Measurement of photosynthetic maximum quantum efficiency and oxygen production

Maximum quantum efficiency (F_v_/F_m_) of dark adapted algae was determined by Pulse Amplitude Modulation (PAM) fluorometry using a diving PAM (Walz, Germany) and a 6 mm diameter fiber optic cable. F_v_/F_m_ measurements were conducted, one hour after the lights turned off automatically. Measurements were taken every evening during the course of the 16d experiment and during the acclimation phase before the experiment, O_2_ concentrations were monitored continuously during the incubations by three 4-channel O_2_ meters (Firesting, Pyroscience, Germany), connected to each chamber with fiber optic cables. Net photosynthesis, respiration, and resulting gross photosynthesis were determined in μM O_2_ h^-1^ relative to organism surface area. In addition, O_2_ consumption was corrected to blank readings from incubation chambers containing only the respective treatment water. For the calculation of daily oxygen evolution rates, the data were extrapolated for a 12 h light and 12 h dark cycle using net photosynthesis and respiration data obtained from one h incubations.

### Quantification of growth rates

Growth was determined only for *H*. *opuntia* as *H*. *macroloba* (a sand dweller) was kept attached to sediment during the course of the incubation, and this would have interfered with the buoyant weight technique [54]. Individual thalli of *H*. *opuntia* (n = 6) were weighed (accuracy: 0.1 mg, Mettler Toledo, USA) in a custom-build buoyant weight set-up with water jacket and seawater of constant temperature (25°C) and salinity (34.5) at the start and end of the experiment. Growth of the organisms was expressed as daily percentage of weight change over the course of the 16 d experiment.

### Quantification of algae pigment content

For the determination of chlorophyll *a* (Chl *a*) content, thalli (n = 6) were frozen to -80°C after incubations. Modified from Schmidt et al. [55] and Vogel and Uthicke [47], apical segments of algae were placed in 15 mL Falcon tubes on ice, and 4 mL of cold ethanol (95% EtOH) was added to extract chlorophyll. To break the inner skeleton of the algae, segments were crushed with a homogenizer, and the samples were heat-shocked in a water bath at 78°C for 5 min. Afterwards, samples were put in a fridge at 4°C for 24 h further extraction, and absorbencies of the supernatant were read at 750 nm and 664 nm on a Powerwave microplate reader (BioTek, USA). Chl*a* content of algal thalli were calculated after equations by Nusch [56] and standardized to segment fresh weight.

### Quantification of carbon and nitrogen contents in algal tissues

Apical segments of *H*. *opuntia* and *H*. *macroloba* (n = 6) were dried at 60°C for 48 h immediately after the incubation. Segments were ground to homogenous powder with a mortar and pestle, and pre-balanced amounts of the homogenate (MX-5 microbalance accuracy: 1μg, Mettler Toledo, Germany) were analysed for total nitrogen and total carbon content on a Euro EA elemental analyser (HEKAtech, Germany). Organic carbon was determined on pre-weighted samples after removing C_inorg_ by acidifying the sample with 300 μL concentrated (37%) HCl. Inorganic carbon content was calculated by subtracting C_org_ from C_tot_.

### Statistical analyses

All data are given with averages and standard deviation in parentheses. We tested all individual responses for significant differences of the individual and combined treatments using a Two-Way-ANOVA. Data was tested for normality using the Shapiro-Wilk test and for equal variance using the Levene median test. Data of net and gross photosynthesis were log_10_ transformed to meet the criteria of equal variance. The Two Way ANOVA was performed with the treatments DIC and DOC as fixed factors and “aquarium” as nested factor. In case the interaction term was significant, differences between individual treatment combinations were evaluated, using a Pairwise Multiple Comparison Procedure (Holm-Sidak method). To further elucidate correlations between response variables, a Pearson product moment correlation was performed and a correlation based Principal Component Analysis (PCA) of all grouped response variables was used to identify covariance among response variables. We considered strong correlation (loadings) of the PC and the response variable to be higher than 0.69. In addition the correlation of NO_x_ fluxes, PO_4_ fluxes and DOC fluxes were plotted in relation to the respiration rate for *H*. *opuntia* and the Pearson product moment correlation was given. All statistical analyses were conducted using SigmaPlot 12.5, NCSS Statistical Software and Statistika 12.0.

## Results

### Effects of DIC

**Fig 1:**
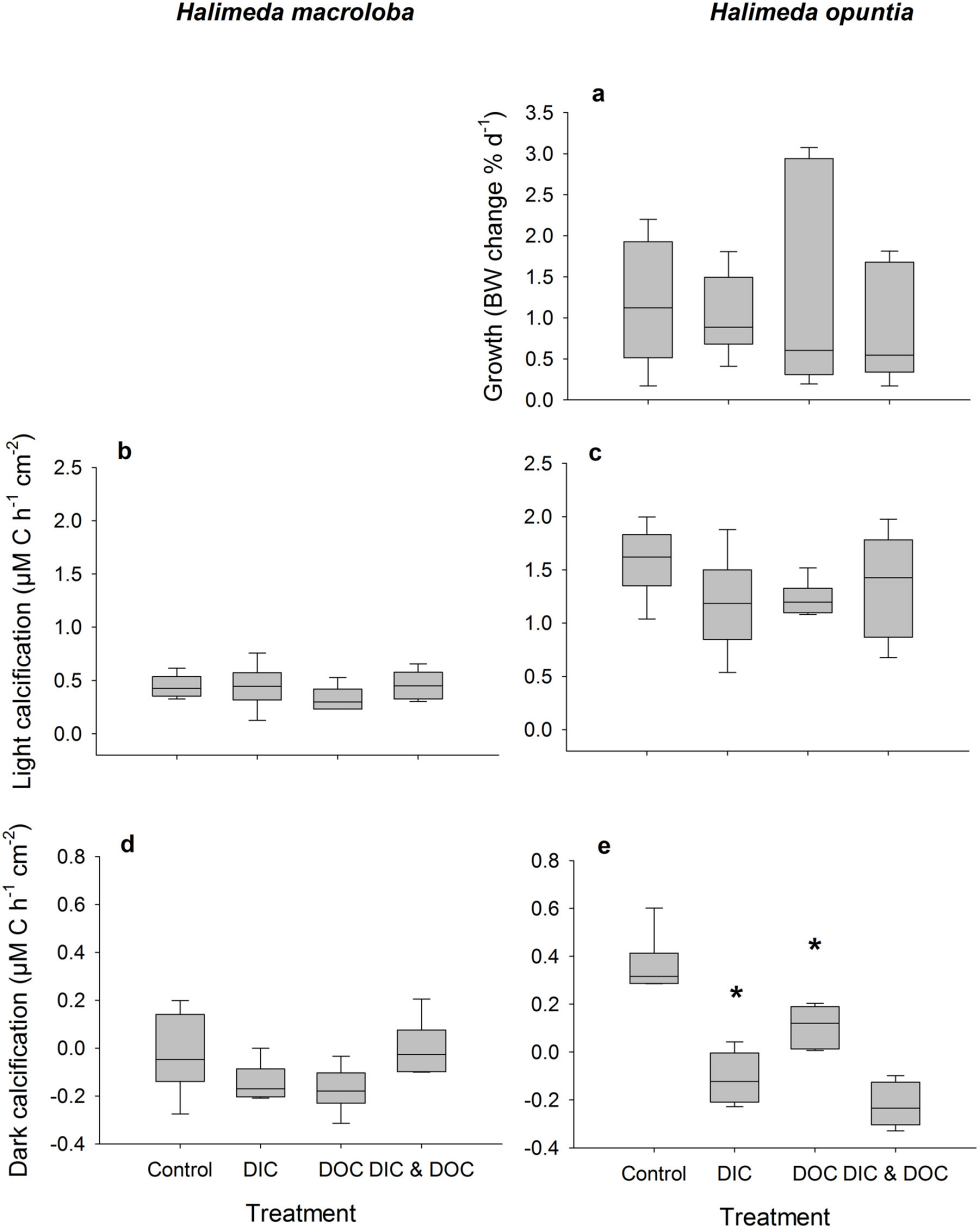
Growth parameters as treatment response after experimental duration of 16 d. Treatments were controls (ambient DIC and DOC), DIC (high DIC and ambient DOC), DOC (ambient DIC and high DOC) and DIC &DOC. Growth is expressed as % change of buoyant weight (a) of *Halimeda opuntia* (n = 6). Calcification during light (b, c; 150 μE m^-2^ s^-1^) and dark condition (d, e) of *Halimeda macroloba* (b, d, n = 6) and *Halimeda opuntia* (c, e, n = 6) measured via alkalinity anomaly technique and standardized to surface area. Significant differences compared to controls (p < 0.05) are marked with an asterisk. Box plots show the median as well as the upper and lower quartile. Whiskers indicate the 95 percentile.

**Fig 2:**
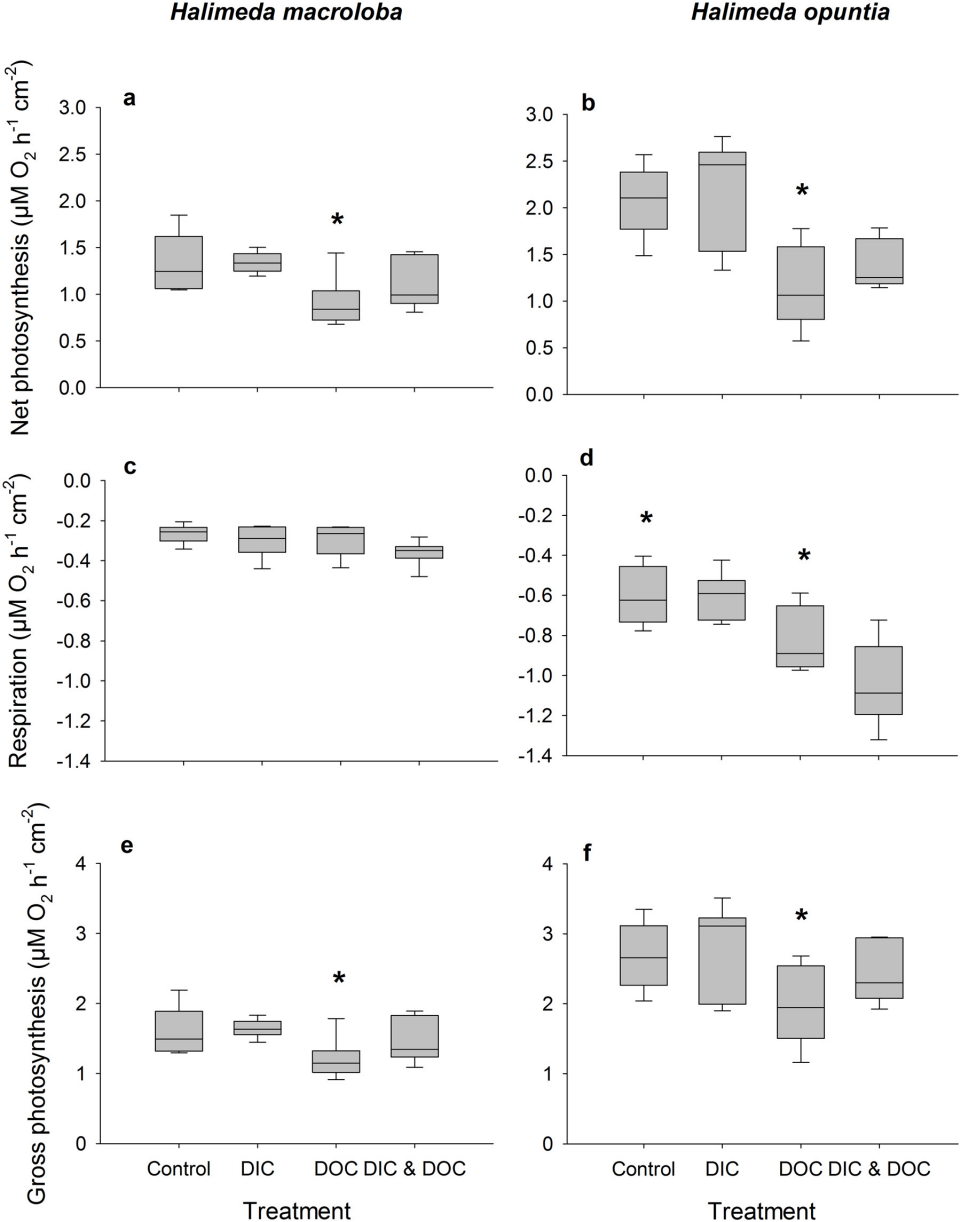
Oxygen fluxes as treatment response. Oxygen fluxes as net photosynthesis (a, b), respiration (c, d) and growth photosynthesis (e, f) of *Halimeda macroloba* (a, c, e; n = 6) and *Halimeda opuntia* (b, d, f; n = 6). Net photosynthesis measured during light (150 μE m^-2^ s^-1^) conditions and respiration during dark condition and standardized to surface area. Significant differences compared to control (p < 0.05) are marked with an asterisk.

**Fig 3:**
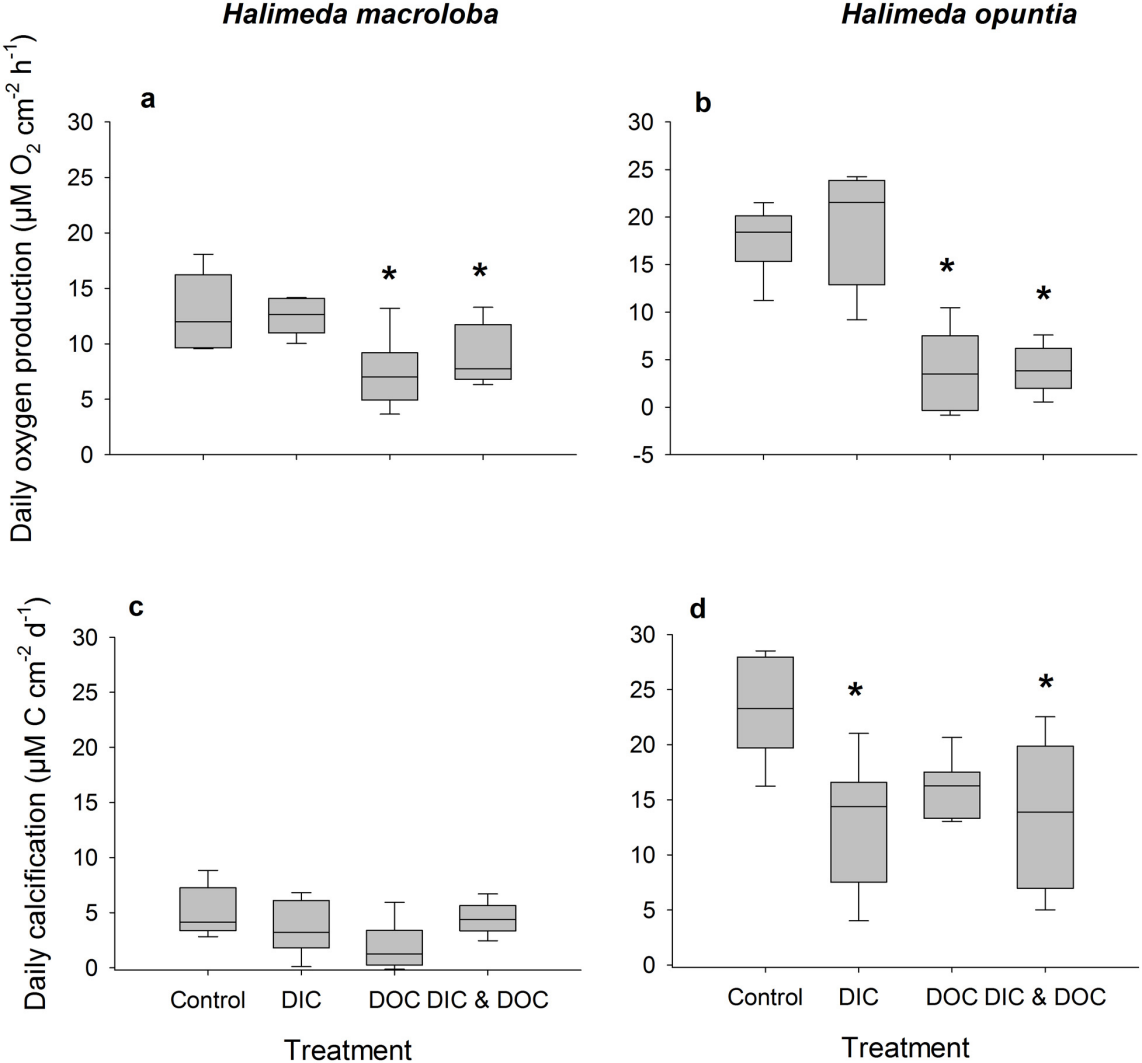
Oxygen production and calcification as treatment response. Oxygen fluxes are calculated over a 12 hour day (150 μE m^-2^ s^-1^) and 12 hour night cycle, of *Halimeda macroloba* (a, n = 6) and *Halimeda opuntia* (b, n = 6). Calcification calculated over a 12 hour day (150 μE m^-2^ s^-1^) and 12 hour night cycle (c, d). Both responses were standardized to surface area. Significant differences compared to control (p < 0.05) are marked with an asterisk.

**Fig 4:**
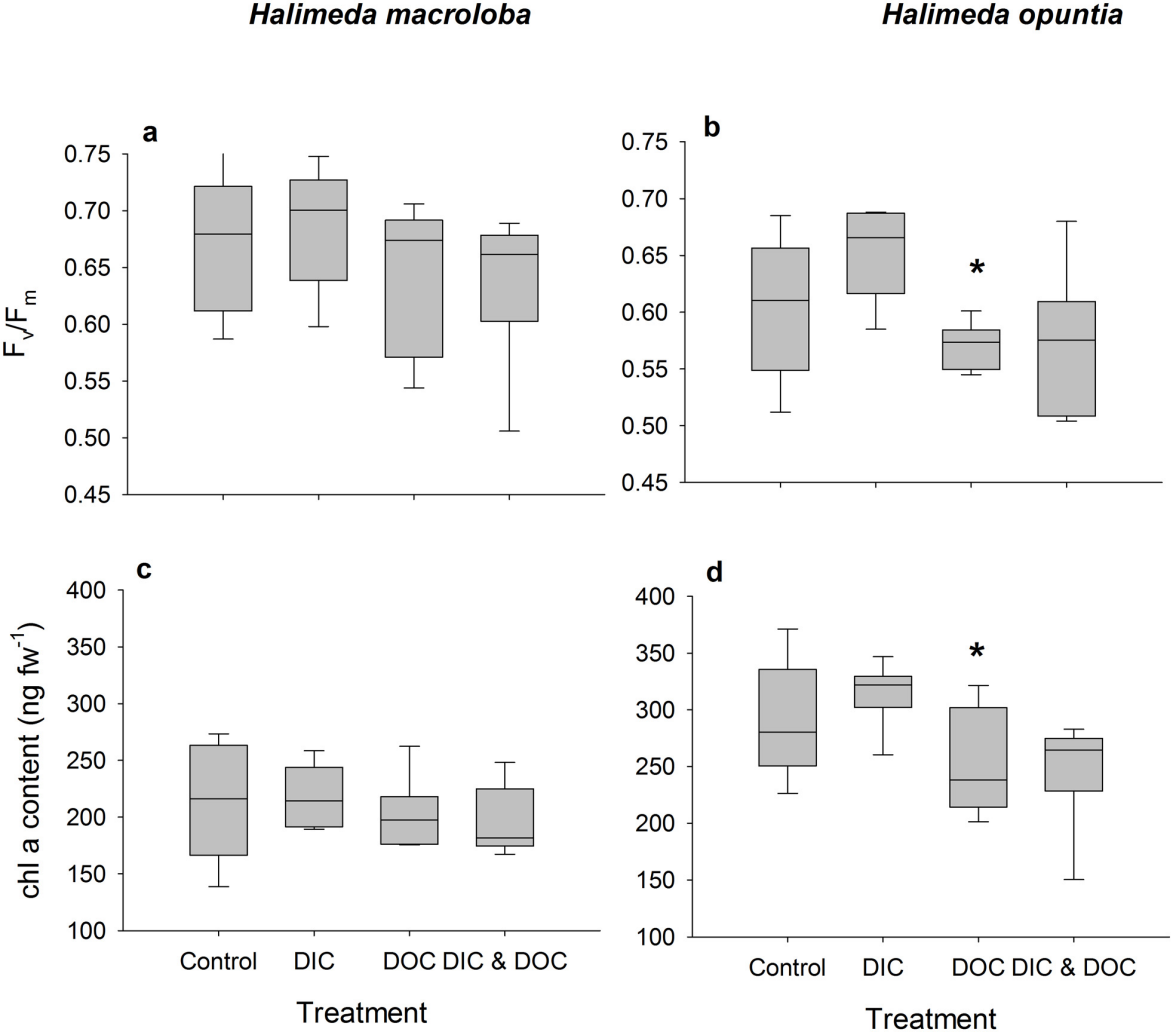
Photosynthetic efficiency and chlorophyll tissue contents as treatment response. Maximum quantum yield (a, b) of dark- adapted individuals *Halimeda macroloba* (a, c; n = 6) and *Halimeda opuntia* (b, d; n = 6). Chlorophyll content (c, d) of both species are standardized to fresh weight. Significant differences compared to control (p < 0.05) are marked with an asterisk.

**Fig 5:**
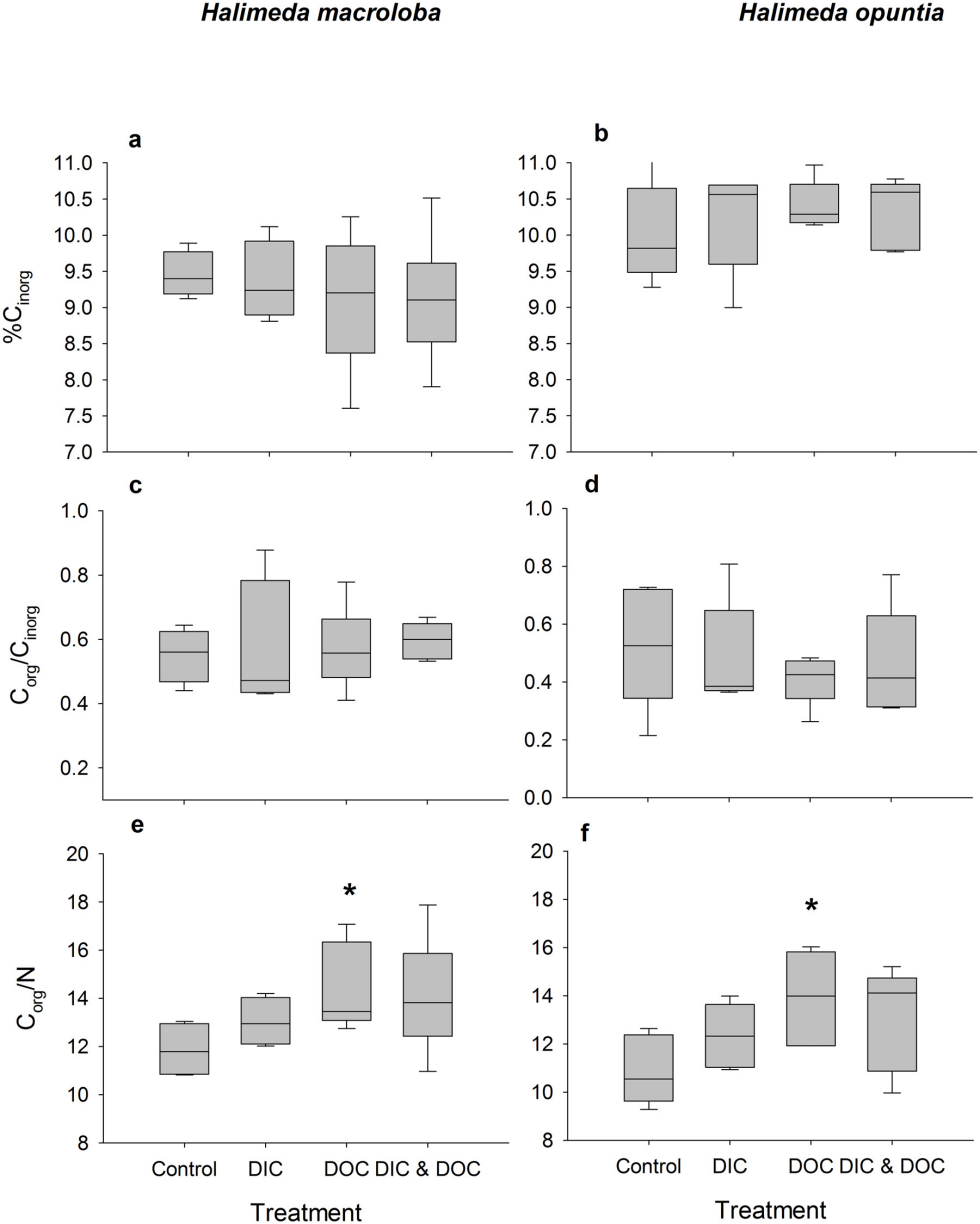
Inorganic and organic C and N algae tissue contents as treatment response. Percent inorganic carbon content (a, b), ratio of organic and inorganic carbon content (c, d) and ratio of organic carbon content to total nitrogen content (e, f) of *Halimeda macroloba* (a, c, e; n = 6) and *Halimeda opuntia* (b, d, f; n = 6).Significant differences compared to control (p < 0.05) are marked with an asterisk.

The biological oxygen demand was relatively low and did not differ between control and high DIC conditions. Elevated DIC conditions (Table 1) did not affect the growth rate of *H*. *opuntia*, or light calcification of both species, but significantly reduced dark calcification rate of *H*. *opuntia* by 130% (Fig 1, p < 0.001, F = 55.05) although there was a tank effect (p = 0.033, F = 3.25). Dark calcification of *H*. *macroloba*, however, was also not affected by high DIC. High DIC did not affect net and gross photosynthesis and respiration (Fig 2), which resulted in no effect on the daily photosynthesis rates of both species (Fig 3). Due to the reduction of dark calcification in *H*. *opuntia* under high DIC, the daily calcification rate was reduced by 45% compared to control conditions (Fig 3) but also showed a tank effect (p = 0.026, F = 3.47). In contrast, high DIC conditions did not affect daily calcification rate of *H*. *macroloba*. Maximum quantum yield as F_v_/F_m_ or Chl*a* content along with inorganic carbon, organic carbon and nitrogen content were not affected in either species (Figs 4 and 5).

**Table 1:** Carbonate system parameters. Values calculated using CO2Calc with total alkalinity and pH_tot_ as input parameters.

Treatment	pH	temp	Salinity	% O_2_Sat	TA	*p*CO_2_	HCO_3_^-^	Ω_Ar_
	[Total]	[°C]	[ppt]		(μmol/kgSW)	(μatm)	(μmol/kgSW)	
**Control**	8.038	25.4	34.4	105.4	2276	402	1776	4.12
	(0.031)	(0.2)	(0.1)	(4.7)	(13)	(11)	(15)	(1.2)
**High DIC**	7.707	25.3	34.4	105.9	2281	996	2011	2.16
	(0.038)	(0.1)	(0.1)	(5.7)	(7)	(26)	(8)	(0.63)
**High DOC**	8.022	25.2	34.4	104.8	2286	430	1792	3.96
	(0.033)	(0.3)	(0.1)	(6.3)	(10)	(10)	(8)	(1.17)
**High DOC**	7.686	25.0	34.4	104.2	2282	1081	2032	2.01
**& DIC**	(0.029)	(0.4)	(0.1)	(5.4)	(17)	(95)	(12)	(0.61)

(n = 3 for TA measurements, n = 10 for pH, temp, salinity and O_2_). Number in parenthesis are standard deviation.

NO_x_ uptake under both dark and light conditions were not affected by high DIC for *H*. *macroloba*, but significantly increased by 45% for *H*. *opuntia* under dark conditions (S1 Fig, p = 0.02, F = 12.76). A similar pattern was observed for NH_4_ uptake where high DIC did not affect the uptake of NH_4_ by *H*. *macroloba* under both light and dark conditions, but significantly increased the uptake of NH_4_ by *H*. *opuntia* under light conditions by 25% (S2 Fig, p = 0.002, F 12.68) including a tank effect (p = 0.007, F = 5.02). Under dark conditions however, NH_4_ uptake of *H*. *opuntia* was reduced by 85% (p = 0.003, F = 18.44). Under high DIC conditions, the PO_4_ uptake of both species was affected. For *H*. *macroloba*, PO_4_ uptake was reduced under light conditions by 17% and increased under dark conditions by 27% compared to the controls (S3 Fig, p = 0.027, F = 7.27). For *H*. *opuntia*, PO_4_ uptake was significantly increased under light conditions by 93% (p = 0.01, F = 4.99) and under dark conditions by 42% compared to the controls where also a tank effect (p = 0.048, F = 2.89) was detected. The DOC uptake rates were not significantly affected by DIC for both species and were only slightly reduced for *H*. *macroloba* by 86% and increased for *H*. *opuntia* by about one order of magnitude under light conditions (S4 Fig).

### Effects of DOC

The biological oxygen demand increased by 115% in the high DOC treatment compared to the controls. High DOC concentrations (Table 1) did not affect the growth of *H*. *opuntia* (Fig 1). Light calcification of both species was also not affected by high DOC, but the dark calcification rate was reduced for *H*. *opuntia* by 70% (Fig 1, p < 0.001, F = 11.42) and a tank effect was detected (p = 0.033, F = 3.25). Net and gross photosynthesis of *H*. *macroloba* were also reduced under the DOC treatment by 32 and 25%, respectively (p = 0.005, F = 14.25 for net photosynthesis, p = 0.009, F = 11.39 for gross photosynthesis) (Fig 2). Similar results were observed for *H*. *opuntia* with a reduction of only net photosynthesis of 45 and 46%, (p = 0.002, F = 20.5). Respiration was only affected by the high DOC treatment for *H*. *opuntia* and resulted in an increased respiration of 37% (p = 0.004, F = 16.38). As a result of the reduced photosynthesis rates of both species under high DOC, daily oxygen production rates of both species were significantly reduced by 78% for *H*. *macroloba* (p 0.004, F = 15.85) and by 32% from *H*. *opuntia* (Fig 3, p < 0.001, F = 57.97). In contrast, the daily calcification rate of both algal species was not affected (Fig 3).

Chl *a* content was reduced by 13%, for *H*. *opuntia* only (Fig 4, p = 0.01, F = 8.16). For the elemental composition no significant effects of the DOC treatment was observed, the C_org_/N ratio was slightly increased for both species under the high DOC treatment compared to the controls (Fig 5). For *H*. *macroloba*, C_org_/N was increased by 28% from and for *H*. *opuntia* by 21% (Fig 5). NO_x_ uptake of both algae species was increased only under dark conditions by 51% for *H*. *macroloba* (p = 0.0027, F = 18.27) and by 117% for *H*. *opuntia* (S1 Fig, p = 0.002, F = 3.28). Ammonia uptake rates of both species in light and dark conditions were not affected by elevated DOC concentrations (S2 Fig). PO_4_ fluxes of *H*. *opuntia* only increased by 45% during dark (S3 Fig, p = 0.03, F = 7.42).

DOC uptake rates for *H*. *macroloba* and *H*. *opuntia* were affected under both dark and light conditions and increased by 458% from (p = 0.032, F = 6.76) in the light and by 369% (p = 0.007, F = 1.17) in the dark for *H*. *macroloba*. For *H*. *opuntia*, DOC uptake rates were highly increased by 6854 and 331% (p < 0.001, F = 38.88 and p < 0.001, F = 35.59) (S4 Fig) but a tank effect was detected (p = 0.016, F = 4.00).

### Effects of the combined treatment

The biological oxygen demand was significantly increased by the combined treatment compared to the DOC treatment by another 81%. In the combined treatment, response variables of the individual treatments showed additive effects as well as synergistic effects. For the dark calcification rate of *H*. *opuntia*, negative effects of both DOC and DIC treatment alone were additive and resulted in further decrease of dark calcification by another 30% compared to the DIC and 100% compared to the DOC treatment (Figs 1 and 3). Similarly, NO_x_ uptake of *H*. *opuntia* under dark conditions increased by 90% compared to the DIC treatment and by 48% compared to the DOC treatment (S1 Fig). This effect was synergistic, leading to a higher effect as the sum of both individual treatment effects alone. Antagonistic effects and increase of NH_4_ uptake was observed for *H*. *opuntia* under dark conditions where the uptake rate was increased in the presence of both, high DIC and DOC concentrations by 236% (S2 Fig). PO_4_ uptake in the dark showed additive effects of the individual treatments which lead to a higher PO_4_ uptake rate of 33% compared to the DIC treatment and 30% compared to the DOC treatment alone (S3 Fig). DOC uptake rates of *H*. *opuntia* in the dark were 390% higher than in the DIC treatment alone and 90% higher than in the DOC treatment resulting in an increase of 420% compared to the control conditions (S4 Fig).

### Connection between the different response variables

**Fig 6:**
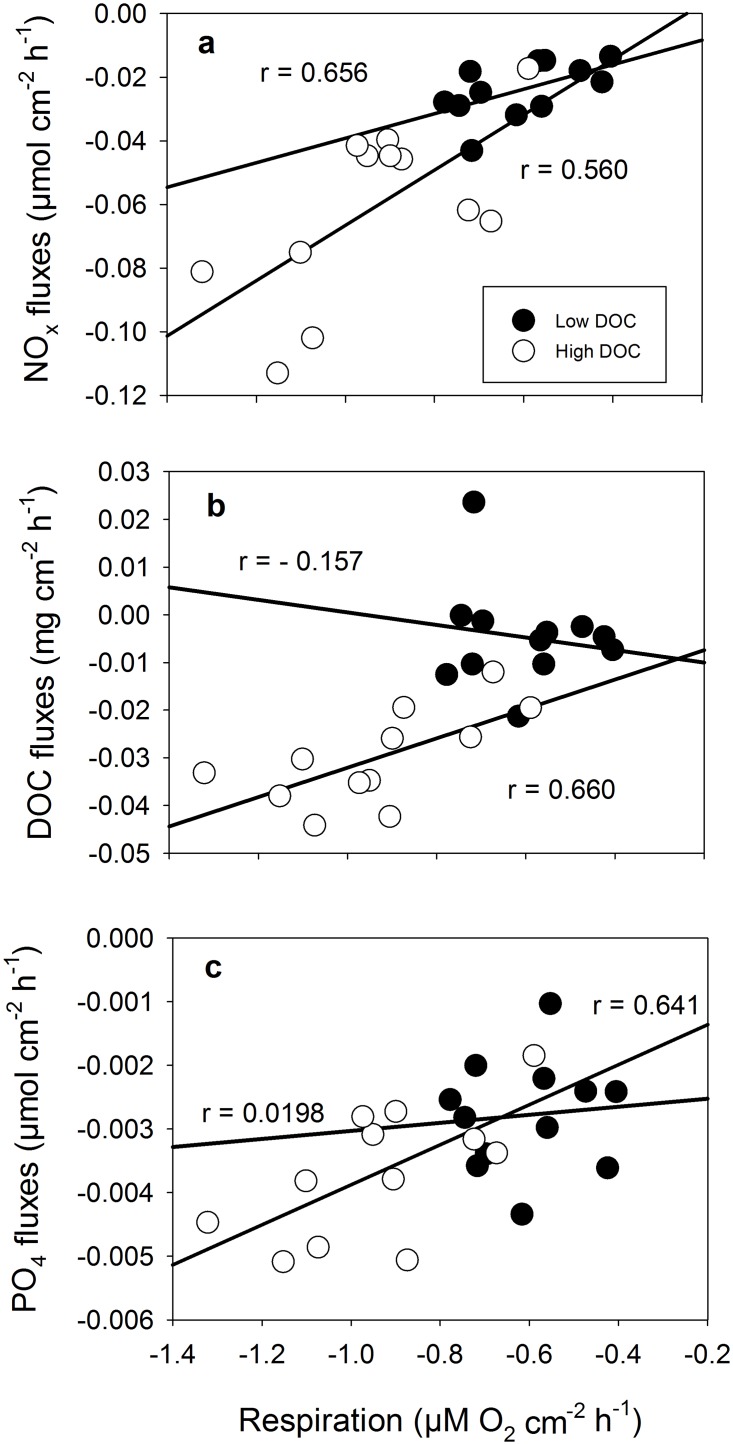
Correlation of response variables in for different DOC concentrations of *Halimeda opuntia*. (a) NO_x_ fluxes, (b) DOC fluxes and (c) PO_4_ fluxes versus respiration at low (filled circles) and high (empty circles) DOC concentration. Pearson’s correlation coefficient (r) is given for each relationship for low and high DOC concentration.

**Fig 7:**
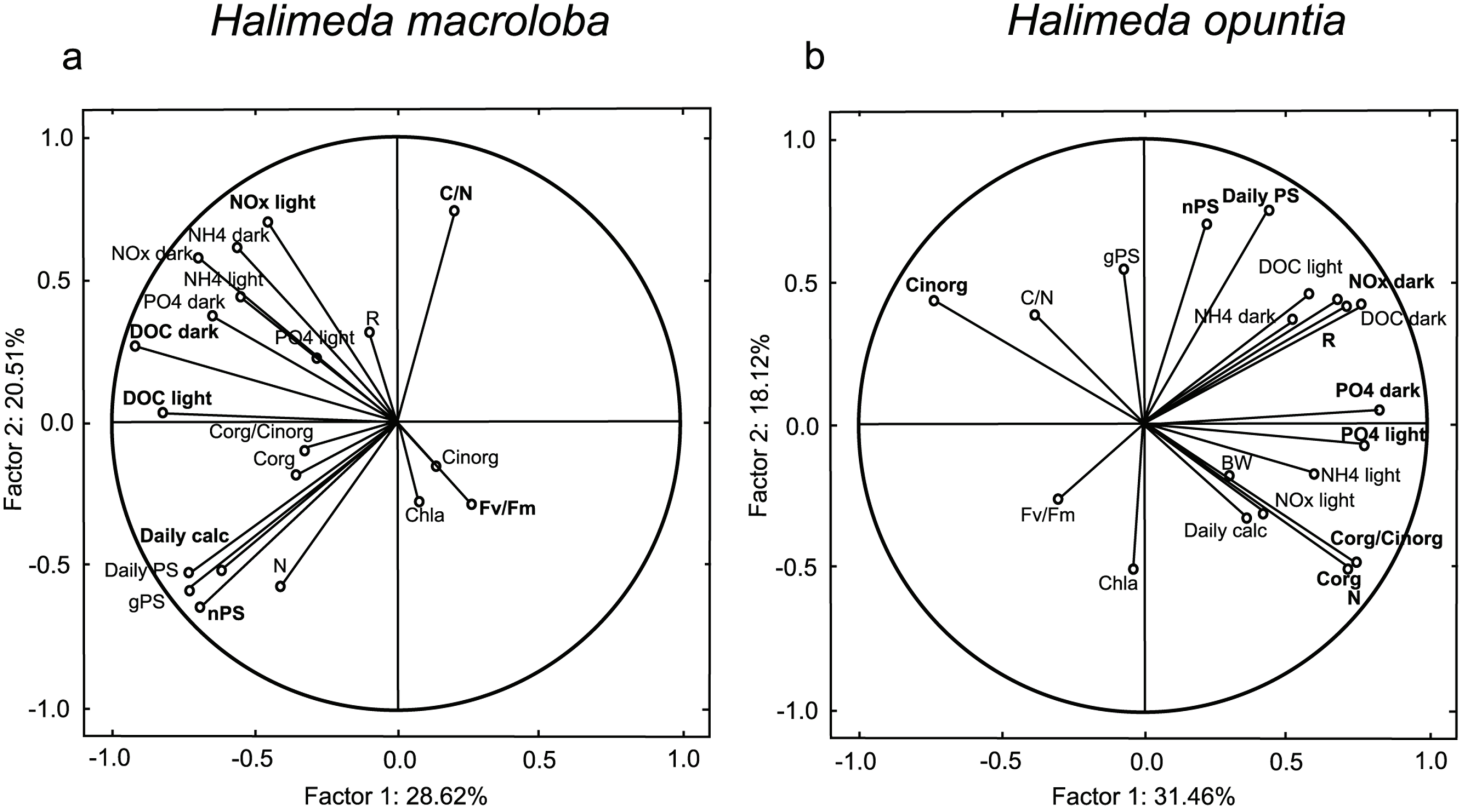
Principal components (PC) 1 and PC 2 of *Halimeda macroloba* (a) and *H*. *opuntia* (b) for all response variable measured. Values were obtained using a correlation based PCA and values considered to weigh heavy (>0.69) are marked in bold.

The correlation of nutrient and DOC fluxes against the respiration rate of *H*. *opuntia* showed that with increasing respiration rates, NO_x_, PO_4_ and DOC uptake rates increased under high DOC concentrations (Fig 6). For the uptake of DOC (Fig 6b) as well as PO_4_ (Fig 6c) no significant correlation to the respiration rate was detected under low DOC conditions but NO_x_ uptake rates showed that with increasing respiration rates less NO_x_ is taken up under low DOC conditions compared to high DOC conditions. The PCA of all data from *H*. *macroloba* combined for the DIC treatment and DOC treatment showed that 29 and 21% of the total variance among the response variables could be explained by principal component (PC) 1 and PC2, respectively (Table 2, Fig 7a). The highest correlations (factor loadings) with PC 1 were found in DOC fluxes in the dark and light, the daily calcification rate and net photosynthesis. On PC 2, only the carbon to nitrogen ratio and the total nitrogen content made a significant contribution. For *H*. *opuntia*, 21 and 18% of the total variance among the response variables was found in PC1 and PC2, respectively (Table 3, Fig 7b). The highest factor loadings on PC 1 were found in PO_4_ fluxes under dark and light conditions, NO_x_ fluxes in the dark as well as total nitrogen and organic carbon content and inorganic carbon content and the respiration rate. For PC 2, net photosynthesis and the daily photosynthesis rate made a significant contribution.

**Table 2:** Factor loadings of principal components (PC) 1 (28.62% total variance) and PC 2 (20.51% total variance) for all response variable measured for *Halimeda macroloba*. Values were obtained using a correlation based PCA and values considered to weigh heavy (>0.69) are marked in bold.

Response variable	PC 1	PC 2
Net photosynthesis	**-0.73**	-0.59
Respiration	-0.09	0.30
Grossphotosynthesis	-0.68	-0.64
Daily photosynthesis rate	-0.61	-0.51
Daily calcification rate	**-0.74**	-0.52
Cinorg	0.13	-0.15
Corg/inorg	-0.31	0.09
C/N	0.19	**0.73**
Corg	0.34	-0.17
N	-0.41	-0.56
NO_x_ flux light	-0.44	**0.71**
NO_x_ fluxdark	-0.68	0.57
NH_4_ flux light	-0.56	0.44
NH_4_ fluxdark	-0.56	0.61
PO_4_ flux light	-0.29	0.23
PO_4_ fluxdark	-0.65	0.38
DOC flux light	**-0.81**	0.04
DOC fluxdark	**-0.91**	0.27
Photochemical efficiency (F_v_/F_m_)	0.25	-0.27
Chl *a* content	0.07	-0.27

**Table 3:** Factor loadings of principal components (PC) 1 (31.46% of total variance) and PC 2 (18.12% of total variance) for all response variable measured for *Halimeda opuntia*. Values were obtained using a correlation based PCA and values considered to weigh heavy (> 0.69) are marked in bold.

Response variable	PC 1	PC 2
Net photosynthesis	0.21	**0.70**
Respiration	**0.70**	0.42
Gross photosynthesis	-0.07	0.53
Daily photosynthesis rate	0.43	**0.75**
Daily calcification rate	0.36	-0.32
Cinorg	**-0.73**	0.43
Corg/inorg	**0.73**	-0.47
C/N	-0.37	0.38
Corg	**0.71**	-0.48
N	**0.71**	-0.49
NO_x_ flux light	0.41	-0.30
NO_x_ flux dark	**0.74**	0.42
NH_4_ flux light	0.58	-0.15
NH_4_ flux dark	0.52	0.36
PO_4_ flux light	**0.76**	-0.06
PO_4_ flux dark	**0.82**	0.06
DOC flux light	0.57	0.46
DOC flux dark	0.67	0.43
Photochemical efficiency (F_v/_F_m_)	-0.29	-0.25
Chl *a* content	-0.04	-0.50
Growth	0.30	-0.19

## Discussion

### Experimental settings

The treatment values reached during the incubation experiment for both high DIC and DOC, as well as the controls were within natural ranges. For the DIC treatment, the resulting *p*CO_2_ of 996 μatm and 1081 μatm (Table 1) for the combined treatment is well represented in the middle of the most recent RCP’s for the year 2100 that predict ranges of 850 (RCP 6.0) to 1370 (RCP 8.5) μatm CO_2_ [3]. Due to the use of natural seawater, the resulting natural A_T_ of 2280 and a saturation of aragonite of 4.1 under the control conditions well represents present day aragonite saturation states in coral reefs. Due to the higher CO_2_ content of the DIC treatment, the saturation was reduced by 50% resulting in Ω_Arag_ = 2.0, well above under-saturated conditions, but below thresholds for projected reef growth [57]. Hence, the manipulated conditions of ocean acidification resemble well the potential OA conditions of the year 2100 in both CO_2_ content as well as Ω_Arag_.

For the DOC treatment, comparable data from other studies are scarce, however the resulting treatment concentration of 294 ± 506 μmol L^-1^ (Table 1) lies within or even below those values reported from other studies that used labile sugars such as glucose and lactose [26,30] and is in the lower range of values already described for natural reef settings [29]. When compared to reported DOC concentrations in the GBR between 66 μmol L^-1^ [31] and 583 μmol L^-1^ (7 mg L^-1^) [32], the DOC concentration in our study (294 to 800 μmol L^-1^) are well within described ranges for DOC concentrations. Although in natural reef settings, the DOC pool might be composed of a multitude of substances, the part of labile DOC used in this study reflects upper ranges of concentrations found under disturbed reef conditions [29]. The average treatment level, therefore was low compared to studies and environmental settings named above.

### Effects of DIC

Our results show that under elevated DIC conditions, the process of calcification is the most influenced, while primary production and photosynthetic performance stay relatively stable. We could not find an effect of high DIC on the calcification or photosynthesis of *H*. *macroloba*, but *H*. *opuntia* showed a decreased daily calcification rate, primarily due to the reduction in the dark calcification rate. This is in line with recent research on *H*. *opuntia* that showed a reduction in calcification of *H*. *opuntia* under OA conditions, but no reduction of calcification of a related *Halimeda* species (*Halimeda taenicola)* [22]. Other studies however did not find an effect of OA on the calcification of *H*. *opuntia* [19], but showed that inorganic carbon content was reduced. This could indicate an effect of reduced calcification. Another study however found high reduction in calcification, photosynthesis, and photochemical efficiency of *H*. *macroloba* under OA conditions [20]. One explanation for the different responses towards OA we observed for *H*. *macroloba* and *H*. *opuntia* could be the different morphology as already discussed for other species comparisons [18,20,22]. In addition we detected a tank effect for this parameter measured which could have altered the effect of DIC and DOC on calcification and reduced or increased the effect we measured. *H*. *macroloba* exhibits lower photosynthetic and calcification rates compared to *H*. *opuntia*. The demand for energy to keep the calcification process high is therefore likely higher in *H*. *opuntia*. Hence, respiration under dark conditions is also likely to be higher which may enhance effects of DIC due to the addition of respiratory CO_2_ and thereby explain the observed dissolution under dark conditions. Observations of flume experiments with *Halimeda* showed similar results, where under ambient CO_2_ conditions, calcification during day-time was balanced out during night-time [58]. In order to maintain high photosynthetic activity, more inorganic nutrients may also be taken up. This may explain the higher NO_x_ as well as NH_4_ uptake observed during dark conditions. This effect has not been described before, but evidence from nutrient enrichment experiments suggest a better performance of *H*. *opuntia* under inorganic nutrient-enriched OA conditions [19].

### Effects of DOC

Compared to the more species-specific effect of high DIC, elevated DOC had a negative effect on both algae species. To our knowledge this is the first study that revealed effects of organic carbon enrichment on macroalgae. We were able to show that elevated DOC conditions have a strong negative impact on the photosynthetic performance of both *Halimeda* species and on dark calcification for *H*. *opuntia*. A species-specific trend appears under elevated DOC conditions as *H*. *opuntia* also suffers from a reduction in photochemical efficiency and Chl *a* content. Together with the reduction in photosynthesis, the elevated DOC conditions may favor non-beneficial or even harmful bacterial growth on the algae itself as also observed by Kuntz et al. [26] on corals. This is supported by higher uptake rates of DOC by bacteria under the high DOC treatment, which may lead to a reduction of the photosynthetic apparatus via proteolysis from bacterial-derived enzymes and also co-competition for space and nutrients and reduction of the nutrient/gas exchange over the algae tissue [59]. Net uptake of DOC most likely took place by bacteria, as *Halimeda* has been shown to release very little DOC or show net uptake compared to rapid uptake of labile DOC by bacteria [35,36]. The bacteria were introduced with the algae to the experimental tanks, were not removed, and therefore showed a strong treatment response. Less filtration of the flow through water could have increased the overall O_2_ consumption, however under high flowthrough rates, accumulation is assumed to be minimal. As different studies have shown, *Halimeda* can also exhibit net release of DOC [27,60] which does not lead to the loss of primary production as observed in the present study. The composition of DOC however is very different from pure glucose as used in this study and my favour its own, beneficial bacterial community [39,61]. The different metabolic rates of the algae may explain the different intensity of reaction towards the treatment in which the more structured morphology of *H*. *opuntia* may favor more bacterial growth due to higher substrate availability. This is supported by the correlation analysis of the response variables for this species which shows that as respiration rates increase, the uptake rates of NO_x_, PO_4_ and also DOC increase under high DIC conditions (Fig 6). Under a future scenario where elevated DOC from river runoff concentrations are connected to elevated N and P concentration, a N and P surplus may in low concentrations benefit the algal fitness and compensate for a loss in primary productivity. However, nutrient uptake under high N and P conditions also increases [18], which leads to a higher demand for energy which may not be compensated by the negative effect of high DOC concentrations on the primary production of the alga. In addition, elevated P and N concentrations may favour fast growing, turf and fleshy algae that are in direct competition with *Halimeda* [60].

### Effects of the combined treatment

Under the combination of both treatments, this study observed additive effects for dark calcification, respiration, and DOC fluxes, indicating that exposure to both factors increases the stress on the algae. As dark calcification further decreased under the presence of both factors compared to the individual factors, it is likely that under future conditions when both factors are present, the investigated algae species will experience loss in total calcification. Over the course of the relatively short experiment, however, we could not observe an interactive negative effect on calcification. The positive interaction, leading to a synergism in NO_x_ uptake indicates that physiological responses are not always additive, but depend on the level of the other factor present. The observed additive effect on DOC uptake may be explained by DOC-facilitated bacterial growth on the algae [38] which was further enhanced under the high DIC treatment. An increased degradation of polysaccharides has been described under elevated DIC conditions [61] which could explain the increase of DOC uptake rates under the combined treatment compared to the high DOC treatment alone. These changes in microbial degradation processes have been assigned to altered catabolic enzymatic activities [61] while rates of primary productions remain stable [62]. This is also in line with the observations of the biological oxygen demand which also showed an interactive effect of the combined treatment and further increased compared to the high DOC treatment, but did not change under high DIC concentrations alone.

### Connection and correlation between the different response variables

We found that many response variables were highly correlated. Daily calcification, photosynthesis, C_inorg_, C_org_, Chl *a* content and photochemical efficiency had a high contribution on the principal components (PC) of both algal species, indicating that the responses towards both environmental factors were strongly associated with those variables. This is in line with our observations that the photosynthetic performance under elevated DOC conditions and the calcification process under elevated DIC conditions were primarily affected.

### Ecological perspective and outlook

The present study showed that there are strong species-specific responses by macroalgae of the genus *Halimeda* towards both applied environmental factors. Depending on the metabolic activity, expressed as the respiration rates, the effect of high and combined DIC and DOC exposure on *H*. *opuntia* was much more pronounced than on *H*. *macroloba*. This is the first study that indicates potential reduction of primary production for algae-dominated benthic communities under high DOC concentrations. Under the presence of both factors, additive as well as synergistic effects further reduced primary production and increased nutrient uptake rates. For further assessment of carbon production rates in coral reefs, not only different factors have to be considered, but also their interactive physiological effects.

Compared to corals, *Halimeda* spp. showed similar reactions towards OA, especially expressed as reduced dark calcification rates. Primary production however was unaffected. Under elevated DOC conditions, no spread of pathogens or bleaching was observed as shown for corals [26,30] but a loss in productivity indicates severe negative effects on their physiology. In the perspective of future phase shift towards algae dominated reefs, the contribution of *Halimeda* towards primary production or carbon accretion may be reduced under the combination of both high DIC and DOC conditions and might provide less carbonate to future buffer capacities of coral reefs, independent of its different morphological structure and different contribution to reef internal carbon cycling [63]. For *Halimeda* dominated reefs [16,64], a future reduction of primary production under elevated DOC concentrations may lead to an additional decrease of carbon accretion where high levels of DIC are present. This might drive a shift of species with heavily calcified (in this case *H*. *opuntia*) domination to a less calcified, less productive species (in our case *H*. *macroloba*) which may have severe impacts on the overall carbon budget of reefs, carbon fixation rates and future buffer capacities against further acidification.

## Supporting Information

S1 Fig NO_x_ fluxes as treatment response. Fluxes are calculated from light (150 μE m^-2^ s^-1^) (a, b) and dark incubations (c, d) of *Halimeda macroloba* (a, c; n = 6) and *Halimeda opuntia* (a, b; n = 6).Significant differences compared to control (p < 0.05) are marked with an asterisk.
(TIF)

S2 Fig NH_4_ fluxes as treatment response. Fluxes calculated from light (150 μE m^-2^ s^-1^) (a, b) and dark incubations (c, d) of *Halimeda macroloba* (a, c; n = 6) and *Halimeda opuntia* (a, b; n = 6).Significant differences compared to control (p < 0.05) are marked with an asterisk.
(TIF)

S3 Fig PO_4_ fluxes as treatment response. Fluxes calculated from light (150 μE m^-2^ s^-1^) (a, b) and dark incubations (c, d) of *Halimeda macroloba* (a, c; n = 6) and *Halimeda opuntia* (b, d; n = 6).Significant differences compared to control (p < 0.05) are marked with an asterisk.
(TIF)

S4 Fig DOC fluxes as treatment response. Fluxes calculated from light (150 μE m^-2^ s^-1^) (a, b) and dark incubations (c, d) of *Halimeda macroloba* (a, c; n = 6) and *Halimeda opuntia* (b, d; n = 6).Significant differences compared to control (p < 0.05) are marked with an asterisk.
(TIF)

S5 Fig DOC concentrations over 12 hours in the high DOC treatment. Time series measurement (08:00 am until 07:00 pm) after addition of 1170 μmol L^-1^ DOC as glucose and a background concentration (unfilled circles) of 76 and 97 μmol L^-1^ DOC. Filled circles indicate sampling points (n = 2) for DOC analysis of the high DOC treatment and unfilled circles of the controls (n = 2 for each point).
(TIF)

S1 Table Statistical results. Results of Two Way ANOVA for both species: Halimeda opuntia and H. macroloba with DIC and DOC as fixed factors and aquaria as nested factor. Significant results are marked bold with an asterisk (*).
(DOCX)
