# Supplementary material for: The Physiological Response of Two Green Calcifying Algae from the Great Barrier Reef towards High Dissolved Inorganic and Organic Carbon (DIC and DOC) Availability
Source: PLoS One. 2015 Aug 12;10(8):e0133596. doi: 10.1371/journal.pone.0133596 (PMC4534297; doi:10.1371/journal.pone.0133596)
Supplement: S1 Table — Results of Two Way ANOVA for both species: Halimeda opuntia and H. macroloba with DIC and DOC as fixed factors and aquaria as nested factor. Significant results are marked bold with an asterisk (*). (DOCX) [file pone.0133596.s006.docx]

**S1 Table. Statistical results.** Results of Two Way ANOVA for both species: *Halimeda opuntia* and *H. macroloba* with DIC and DOC as fixed factors and aquaria as nested factor. Significant results are marked bold with an asterisk (*).

| **Response variable** | **Species** | **Source of variation** | **DF** | **SS** | **MS** | **F-value** | **P-value** |
| --- | --- | --- | --- | --- | --- | --- | --- |
| **Biological oxygen** | - | DIC | 1 | 0.265 | 0.265 | 6.377 | **0.036*** |
| **demand** |  | DOC | 1 | 5.009 | 5.009 | 120.766 | **<0.001*** |
|  |  | DIC x DOC | 1 | 0.394 | 0.394 | 9.510 | **0.015*** |
|  |  | Residual | 9 | 0.332 | 0.0415 |  |  |
|  |  | Total | 11 | 6.000 | 0.545 |  |  |
| **Growth** | *H. opuntia* | DIC | 1 | 0.525 | 0.525 | 0.34 | 0.575 |
|  |  | DOC | 1 | 0.00722 | 0.00722 | 0.00 | 0.947 |
|  |  | DIC x DOC | 1 | 0.118 | 0.118 | 0.08 | 0.788 |
|  |  | Aquaria | 8 | 12.33405 | 1.541756 | 6.16 | **0.003*** |
|  |  | Residual | 12 | 15.336 | 0.767 |  |  |
|  |  | Total | 23 | 15.987 |  |  |  |
| **Light calcification** | *H. macroloba* | DIC | 1 | 0.0216 | 0.0216 | 1.41 | 0.269 |
|  |  | DOC | 1 | 0.0173 | 0.0173 | 1.12 | 0.320 |
|  |  | DIC x DOC | 1 | 0.0213 | 0.0213 | 1.38 | 0.273 |
|  |  | Aquaria | 8 | 0.1232182 | 0.01540228 | 0.59 | 0.764 |
|  |  | Residual | 11 | 0.2851351 | 0.02592138 |  |  |
|  |  | Total | 22 | 0.470 |  |  |  |
|  | *H. opuntia* | DIC | 1 | 0.106 | 0.106 | 0.44 | 0.524 |
|  |  | DOC | 1 | 0.0512 | 0.0512 | 0.21 | 0.656 |
|  |  | DIC x DOC | 1 | 0.423 | 0.423 | 1.76 | 0.220 |
|  |  | Aquaria | 8 | 1.918423 | 0.2398028 | 3.03 | **0.041*** |
|  |  | Residual | 12 | 0.9484203 | 0.07903503 |  |  |
|  |  | Total | 23 | 3.448 |  |  |  |
| **Dark calcification** | *H. macroloba* | DIC | 1 | 0.00379 | 0.00379 | 0.49 | 0.504 |
|  |  | DOC | 1 | 0.0000584 | 0.0000584 | 0.01 | 0.933 |
|  |  | DIC x DOC | 1 | 0.126 | 0.126 | 16.25 | **0.004*** |
|  |  | Aquaria | 8 | 0.062275 | 0.00778 | 0.43 | 0.880 |
|  |  | Residual | 11 | 0.279 | 0.0140 |  |  |
|  |  | Total | 23 | 0.410 |  |  |  |
|  | *H. opuntia* | DIC | 1 | 0.955 | 0.955 | 55.05 | **<0.001*** |
|  |  | DOC | 1 | 0.198 | 0.198 | 11.42 | **<0.001*** |
|  |  | DIC x DOC | 1 | 0.0288 | 0.0288 | 1.66 | 0.233 |
|  |  | Aquaria | 8 | 0.1387564 | 0.0173445 | 3.25 | **0.033*** |
|  |  | Residual | 12 | 0.203 | 0.0101 |  |  |
|  |  | Total | 23 | 1.384 |  |  |  |
| **Daily calcification** | *H. macroloba* | DIC | 1 | 2.087715 | 2.087715 | 0.5 | 0.50 |
|  |  | DOC | 1 | 7.900193 | 7.900193 | 1.88 | 0.206 |
|  |  | DIC x DOC | 1 | 24.57278 | 24.57278 | 5.89 | **0.041*** |
|  |  | Aquaria | 8 | 33.39654 | 4.174568 | 0.86 | 0.57 |
|  |  | Residual | 12 | 57.95209 | 4.829341 |  |  |
|  |  | Total | 23 | 125.9093 |  |  |  |
|  |  |  |  |  |  |  |  |
|  | *H. opuntia* | DIC | 1 | 244.6349 | 244.6349 | 5.21 | **0.0052*** |
|  |  | DOC | 1 | 64.90776 | 64.90776 | 1.38 | 0.273 |
|  |  | DIC x DOC | 1 | 96.85951 | 96.85951 | 2.06 | 0.188 |
|  |  | Aquaria | 8 | 375.6105 | 46.95132 | 3.47 | **0.026** |
|  |  | Residual | 12 | 162.4933 | 13.5411 |  |  |
|  |  | Total | 23 | 944.506 |  |  |  |
| **Net** | *H. macroloba* | DIC | 1 | 0.0142 | 0.0142 | 1.90 | 0.205 |
| **photosynthesis** |  | DOC | 1 | 0.106 | 0.106 | 14.25 | **0.005*** |
|  |  | DIC x DOC | 1 | 0.00876 | 0.00876 | 1.17 | 0.311 |
|  |  | Aquaria | 8 | 0.05976 | 0.00747 | 0.79 | 0.620 |
|  |  | Residual | 12 | 0.113 | 0.00943 |  |  |
|  |  | Total | 23 | 0.302 |  |  |  |
|  | *H. opuntia* | DIC | 1 | 0.183 | 0.183 | 0.82 | 0.39 |
|  |  | DOC | 1 | 4.558 | 4.558 | 20.50 | **0.002*** |
|  |  | DIC x DOC | 1 | 0.0193 | 0.0193 | 0.09 | 0.775 |
|  |  | Aquaria | 8 | 1.778866 | 0.2223582 | 1.38 | 0.295 |
|  |  | Residual | 12 | 1.927572 | 0.160631 |  |  |
|  |  | Total | 23 | 8.467 |  |  |  |
| **Respiration** | *H. macroloba* | DIC | 1 | 0.0300 | 0.0300 | 3.20 | 0.111 |
|  |  | DOC | 1 | 0.0232 | 0.0232 | 2.48 | 0.154 |
|  |  | DIC x DOC | 1 | 0.00296 | 0.00296 | 0.32 | 0.59 |
|  |  | Aquaria | 8 | 0.0749 | 0.00937 | 1.11 | 0.420 |
|  |  | Residual | 12 | 0101 | 0.00884 |  |  |
|  |  | Total | 23 | 0.232 |  |  |  |
|  | *H. opuntia* | DIC | 1 | 0.0714 | 0.0714 | 1.77 | 0.220 |
|  |  | DOC | 1 | 0.662 | 0.662 | 16.38 | **0.004*** |
|  |  | DIC x DOC | 1 | 0.0706 | 0.0706 | 1.75 | 0.223 |
|  |  | Aquaria | 8 | 0.3234091 | 0.040426 | 2.45 | 0.078 |
|  |  | Residual | 12 | 0.1977546 | 0.01.648 |  |  |
|  |  | Total | 23 | 1.325 |  |  |  |
| **Gross** | *H. macroloba* | DIC | 1 | 0.0163 | 0.0163 | 3.67 | 0.092 |
| **photosynthesis** |  | DOC | 1 | 0.0505 | 0.0505 | 11.39 | **0.009*** |
|  |  | DIC x DOC | 1 | 0.00680 | 0.00680 | 1.53 | 0.251 |
|  |  | Aquaria | 8 | 0.0354 | 0.00443 | 0.53 | 0.811 |
|  |  | Residual | 12 | 0.101 | 0.00844 |  |  |
|  |  | Total | 23 | 0.232 |  |  |  |
|  | *H. opuntia* | DIC | 1 | 0.483 | 0.483 | 1.29 | 0.290 |
|  |  | DOC | 1 | 1.746 | 1.746 | 4.65 | 0.063 |
|  |  | DIC x DOC | 1 | 0.164 | 0.164 | 0.44 | 0.53 |
|  |  | Aquaria | 8 | 3.004597 | 0.3755747 | 1.56 | 0.235 |
|  |  | Residual | 12 | 2.890199 | 0.2408499 |  |  |
|  |  | Total | 23 | 8.287 |  |  |  |
| **Daily photosynthesis** | *H. macroloba* | DIC | 1 | 2.126292 | 2.126292 | 0.27 | 0.615 |
|  |  | DOC | 1 | 123.0113 | 123.0113 | 15.85 | **0.004*** |
|  |  | DIC x DOC | 1 | 5.284694 | 5.284694 | 0.68 | 0.433 |
|  |  | Aquaria | 8 | 62.08359 | 7.760448 | 0.91 | 0.54 |
|  |  | Residual | 12 | 102.4736 | 8.53947 |  |  |
|  |  | Total | 23 | 294.9795 |  |  |  |
|  | *H. opuntia* | DIC | 1 | 3.702061 | 3.702061 | 0.17 | 0.69 |
|  |  | DOC | 1 | 1252.035 | 1252.035 | 57.97 | **<0.001*** |
|  |  | DIC x DOC | 1 | 2.307144 | 2.307144 | 0.11 | 0.752 |
|  |  | Aquaria | 8 | 172.7754 | 21.59692 | 1.32 | 0.319 |
|  |  | Residual | 12 | 195.868 | 16.32233 |  |  |
|  |  | Total | 23 | 1626.687 |  |  |  |
| **Maximum quantum yield** | *H. macroloba* | DIC | 1 | 0.000122 | 0.000122 | 0.02 | 0.883 |
|  |  | DOC | 1 | 0.00859 | 0.00859 | 0.13 | 0.729 |
|  |  | DIC x DOC | 1 | 0.000683 | 0.000683 | 1.63 | 0.238 |
|  |  | Aquaria | 8 | 0.0422686 | 0.005283 | 1.77 | 0.179 |
|  |  | Residual | 12 | 0.0781 | 0.00390 |  |  |
|  |  | Total | 23 | 0.0875 |  |  |  |
|  | *H. opuntia* | DIC | 1 | 0.00375 | 0.00375 | 1.510 | 0.307 |
|  |  | DOC | 1 | 0.0198 | 0.0198 | 7.990 | 0.332 |
|  |  | DIC x DOC | 1 | 0.00336 | 0.00336 | 1.354 | **0.036*** |
|  |  | Aquaria | 8 | 0.2516267 | 0.003145333 | 1.54 | 0.241 |
|  |  | Residual | 12 | 0.0497 | 0.00248 |  |  |
|  |  | Total | 23 | 0.0766 |  |  |  |
| **Chlorophyll *a* content** | *H. macroloba* | DIC | 1 | 5.960 | 5.960 | 0.00 | 0.946 |
|  |  | DOC | 1 | 1708.899 | 1708.899 | 1.42 | 0.27 |
|  |  | DIC x DOC | 1 | 188.587 | 188.587 | 0.16 | 0.703 |
|  |  | Aquaria | 8 | 9649.605 | 1206.201 | 0.84 | 0.586 |
|  |  | Residual | 12 | 17201.23 | 1433.436 |  |  |
|  |  | Total | 23 | 28754.283 |  |  |  |
|  | *H. opuntia* | DIC | 1 | 668.851 | 668.851 | 0.331 | 0.571 |
|  |  | DOC | 1 | 16474.195 | 16474.195 | 8.163 | **0.010*** |
|  |  | DIC x DOC | 1 | 1278.336 | 1278.336 | 0.633 | 0.435 |
|  |  | Aquaria | 8 | 18151.98 | 2268.997 | 1.23 | 0.362 |
|  |  | Residual | 12 | 40363.234 | 2018.162 |  |  |
|  |  | Total | 23 | 58784.615 |  |  |  |
| **Inorganic C** | *H. macroloba* | DIC | 1 | 0.00974 | 0.00974 | 0.02 | 0.879 |
|  |  | DOC | 1 | 0.416 | 0.416 | 1.05 | 0.335 |
|  |  | DIC x DOC | 1 | 0.0149 | 0.0149 | 0.04 | 0.851 |
|  |  | Aquaria | 8 | 3.168803 | 0.3961004 | 0.54 | 0.8 |
|  |  | Residual | 8 | 9.047 | 0.565 |  |  |
|  |  | Total | 19 | 9.484 |  |  |  |
|  | *H. opuntia* | DIC | 1 | 0.0458 | 0.0458 | 0.141 | 0.711 |
|  |  | DOC | 1 | 0.396 | 0.396 | 1.223 | 0.283 |
|  |  | DIC x DOC | 1 | 0.0919 | 0.0919 | 0.284 | 0.601 |
|  |  | Aquaria | 8 | 3.431844 | 0.4289804 | 1.79 | 0.191005 |
|  |  | Residual | 10 | 5.826 | 0.324 |  |  |
|  |  | Total | 21 | 6.388 |  |  |  |
| **Organic C / Inorganic C** | *H. macroloba* | DIC | 1 | 0.00166 | 0.00166 | 0.14 | 0.715 |
|  |  | DOC | 1 | 0.00361 | 0.00361 | 0.31 | 0.592 |
|  |  | DIC x DOC | 1 | 0.000202 | 0.000202 | 0.02 | 0.898 |
|  |  | Aquaria | 8 | 0.009260 | 0.001576 | 0.60 | 0.760 |
|  |  | Residual | 8 | 0.1554698 | 0.0155 |  |  |
|  |  | Total | 19 | 0.2538606 |  |  |  |
|  | *H. opuntia* | DIC | 1 | 0.000787 | 0.000787 | 0.0265 | 0.873 |
|  |  | DOC | 1 | 0.0202 | 0.0202 | 0.678 | 0.421 |
|  |  | DIC x DOC | 1 | 0.0107 | 0.0107 | 0.360 | 0.556 |
|  |  | Aquaria | 8 | 0.2579519 | 0.03224 | 1.16 | 0.405 |
|  |  | Residual | 10  0 | 0.536 | 0.0298 |  |  |
|  |  | Total | 21 | 0.567 |  |  |  |
| **Organic C / N** | *H. macroloba* | DIC | 1 | 1.027 | 1.027 | 0.334 | 0.574 |
|  |  | DOC | 1 | 15.020 | 15.020 | 5.01 | 0.055 |
|  |  | DIC x DOC | 1 | 2.381 | 2.381 | 0.79 | 0.399 |
|  |  | Aquaria | 8 | 23.97476 | 2.996845 | 0.89 | 0.563 |
|  |  | Residual | 8 | 50.876 | 3.180 |  |  |
|  |  | Total | 19 | 68.796 |  |  |  |
|  | *H. opuntia* | DIC | 1 | 0.847 | 0.847 | 0.279 | 0.694 |
|  |  | DOC | 1 | 20.905 | 20.905 | 6.883 | 0.077 |
|  |  | DIC x DOC | 1 | 6.586 | 6.586 | 2.169 | 0.288 |
|  |  | Aquaria | 8 | 40.72598 | 5.090747 | 3.65 | 0.03 |
|  |  | Residual | 10 | 54.667 | 3.037 |  |  |
|  |  | Total | 21 | 83.958 |  |  |  |
| **NO_X_ fluxes** | *H. macroloba* | DIC | 1 | 0.0109 | 0.0109 | 0.45 | 0.522 |
| **in light** |  | DOC | 1 | 0.000590 | 0.000590 | 0.002 | 0.880 |
|  |  | DIC x DOC | 1 | 0.0229 | 0.0229 | 0.94 | 0.360 |
|  |  | Aquaria | 8 | 0.194609 | 0.02432612 | 0.41 | 0.897 |
|  |  | Residual | 12 | 0.7206705 | 0.0458 |  |  |
|  |  | Total | 23 | 0.950 |  |  |  |
|  | *H. opuntia* | DIC | 1 | 0.0000290 | 0.0000290 | 0.404 | 0.532 |
|  |  | DOC | 1 | 0.000000493 | 0.000000493 | 0.00685 | 0.935 |
|  |  | DIC x DOC | 1 | 0.000173 | 0.000173 | 2.400 | 0.137 |
|  |  | Aquaria | 8 | 0.000828 | 0.0001034 | 2.03 | 0.129 |
|  |  | Residual | 12 | 0.00144 | 0.0000719 |  |  |
|  |  | Total | 23 | 0.00164 |  |  |  |
| **NO_X_ fluxes** | *H. macroloba* | DIC | 1 | 0.0000778 | 0.0000778 | 1.09 | 0.326 |
| **in dark** |  | DOC | 1 | 0.00129 | 0.00129 | 18.27 | **0.0027*** |
|  |  | DIC x DOC | 1 | 0.000165 | 0.000165 | 2.33 | 0.165 |
|  |  | Aquaria | 8 | 0.000566 | 0.0000707370 | 0.58 | 0.774555 |
|  |  | Residual | 12 | 0.00202 | 0.000101 |  |  |
|  |  | Total | 23 | 0.00356 |  |  |  |
|  | *H. opuntia* | DIC | 1 | 0.00317 | 0.00317 | 12.761 | **0.02*** |
|  |  | DOC | 1 | 0.00827 | 0.00827 | 33.281 | **0.002*** |
|  |  | DIC x DOC | 1 | 0.00122 | 0.00122 | 4.901 | 0.111 |
|  |  | Aquaria | 9 | 0.003032 | 0.00037896 | 2.35 | 0.088 |
|  |  | Residual | 12 | 0.00497 | 0.000248 |  |  |
|  |  | Total | 23 | 0.0176 |  |  |  |
| **NH_4_ fluxes** | *H. macroloba* | DIC | 1 | 0.000283 | 0.000283 | 0.87 | 0.377 |
| **in light** |  | DOC | 1 | 0.000160 | 0.000160 | 0.49 | 0.502 |
|  |  | DIC x DOC | 1 | 0.000830 | 0.000830 | 2.56 | 0.148 |
|  |  | Aquaria | 8 | 0.002587 | 0.00032343 | 0.78 | 0.626 |
|  |  | Residual | 12 | 0.00496 | 0.00042 |  |  |
|  |  | Total | 23 | 0.00881 |  |  |  |
|  | *H. opuntia* | DIC | 1 | 0.00124 | 0.00124 | 12.681 | **0.002*** |
|  |  | DOC | 1 | 0.00000393 | 0.00000393 | 0.0401 | 0.843 |
|  |  | DIC x DOC | 1 | 0.000668 | 0.000668 | 3.54 | 0.1 |
|  |  | Aquaria | 8 | 0.00151 | 0.0001886 | 5.02 | **0.007*** |
|  |  | Residual | 12 | 0.00196 | 0.0000979 |  |  |
|  |  | Total | 23 | 0.00387 |  |  |  |
| **NH_4_ fluxes** | *H. macroloba* | DIC | 1 | 0.00143 | 0.00143 | 18.44 | **0.003*** |
| **in dark** |  | DOC | 1 | 0.00124 | 0.00124 | 15.99 | **0.004*** |
|  |  | DIC x DOC | 1 | 0.00142 | 0.00142 | 18.37 | **0.003*** |
|  |  | Aquaria | 8 | 0.0006213 | 0.0000777 | 0.47 | 0.855 |
|  |  | Residual | 12 | 0.00198 | 0.000154 |  |  |
|  |  | Total | 23 | 0.00670 |  |  |  |
|  | *H. opuntia* | DIC | 1 | 0.0223 | 0.0223 | 6.150 | 0119 |
|  |  | DOC | 1 | 0.0120 | 0.0120 | 3.312 | 0.084 |
|  |  | DIC x DOC | 1 | 0.0201 | 0.0201 | 5.541 | 0.135 |
|  |  | Aquaria | 8 | 0.00587 | 0.0073 | 6.18 | 0.003 |
|  |  | Residual | 12 | 0.0727 | 0.00363 |  |  |
|  |  | Total | 23 | 0.127 |  |  |  |
| **PO_4_ Fluxes** | *H. macroloba* | DIC | 1 | 0.0000153 | 0.0000153 | 7.27 | **0.027*** |
| **in light** |  | DOC | 1 | 0.00000236 | 0.00000236 | 1.11 | 0323 |
|  |  | DIC x DOC | 1 | 0.00000911 | 0.00000911 | 4.32 | 0.071 |
|  |  | Aquaria | 8 | 0.0000169 | 0.00000231 | 2.28 | 0.095 |
|  |  | Residual | 12 | 0.0000111 | 0.00000092 |  |  |
|  |  | Total | 23 | 0.0000549 |  |  |  |
|  | *H. opuntia* | DIC | 1 | 0.00000511 | 0.00000511 | 4.994 | **0.01*** |
|  |  | DOC | 1 | 0.00000341 | 0.00000341 | 7.42 | **0.03*** |
|  |  | DIC x DOC | 1 | 0.0000000523 | 0.0000000523 | 0.0511 | 0.823 |
|  |  | Aquaria | 8 | 0.000003681 | 0.0000004601 | 0.33 | 0.94 |
|  |  | Residual | 12 | 0.0000205 | 0.00000102 |  |  |
|  |  | Total | 23 | 0.0000290 |  |  |  |
| **PO_4_ Fluxes** | *H. macroloba* | DIC | 1 | 0.148 | 0.148 | 4.54 | 0.065 |
| **in dark** |  | DOC | 1 | 0.140 | 0.140 | 4.31 | 0.071 |
|  |  | DIC x DOC | 1 | 0.0611 | 0.0611 | 1.88 | 0.208 |
|  |  | Aquaria | 8 | 0.2602862 | 0.0325 | 1.0 | 0.482 |
|  |  | Residual | 12 | 0.3897141 | 0.0325 |  |  |
|  |  | Total | 23 | 0.999 |  |  |  |
|  | *H. opuntia* | DIC | 1 | 0.00000408 | 0.00000408 | 3.10 | 0.116 |
|  |  | DOC | 1 | 0.00000483 | 0.00000483 | 3.68 | 0.091 |
|  |  | DIC x DOC | 1 | 0.000000107 | 0.000000107 | 0.134 | 0.718 |
|  |  | Aquaria | 8 | 0.00001051 | 0.00000131 | 2.89 | **0.048*** |
|  |  | Residual | 12 | 0.0000160 | 0.000000799 |  |  |
|  |  | Total | 23 | 0.0000250 |  |  |  |
| **DOC fluxes** | *H. macroloba* | DIC | 1 | 0.00151 | 0.00151 | 4.80 | 0.06 |
| **in light** |  | DOC | 1 | 0.00212 | 0.00212 | 6.76 | **0.032*** |
|  |  | DIC x DOC | 1 | 0.000644 | 0.000644 | 2.05 | 0.190 |
|  |  | Aquaria | 8 | 0.00251 | 0.000314 | 1.78 | 0.177 |
|  |  | Residual | 12 | 0.00211 | 0.000176 |  |  |
|  |  | Total | 23 | 0.00890 |  |  |  |
|  | *H. opuntia* | DIC | 1 | 1.039 | 1.039 | 5.19 | 0.052 |
|  |  | DOC | 1 | 4.268 | 4.268 | 38.879 | **<0.001*** |
|  |  | DIC x DOC | 1 | 1.212 | 1.212 | 11.041 | 0.003* |
|  |  | Aquaria | 8 | 1.602184 | 0.200273 | 4.00 | **0.016*** |
|  |  | Residual | 12 | 2.196 | 0.110 |  |  |
|  |  | Total | 23 | 8.714 |  |  |  |
| **DOC fluxes** | *H. macroloba* | DIC | 1 | 0.239 | 0.239 | 1.02 | 0.342 |
| **in dark** |  | DOC | 1 | 3.078 | 3.078 | 13.17 | **0.007*** |
|  |  | DIC x DOC | 1 | 0.0970 | 0.0970 | 0.41 | 0.538 |
|  |  | Aquaria | 8 | 1.87008 | 0.23376 | 1.47 | 0.265 |
|  |  | Residual | 12 | 1.913856 | 0.159488 |  |  |
|  |  | Total | 23 | 7.197 |  |  |  |
|  | *H. opuntia* | DIC | 1 | 0.00000840 | 0.00000840 | 0.0773 | 0.784 |
|  |  | DOC | 1 | 0.00387 | 0.00387 | 35.592 | **<0.001*** |
|  |  | DIC x DOC | 1 | 0.000119 | 0.000119 | 1.093 | 0.308 |
|  |  | Aquaria | 8 | 0.00132 | 0.000166 | 2.35 | 0.087 |
|  |  | Residual | 12 | 0.00217 | 0.000109 |  |  |
|  |  | Total | 23 | 0.00617 |  |  |  |
| **Fv/Fm** | *H. macroloba* | DIC | 1 | 0.0001215 | 0.0001215 | 0.02 | 0.883 |
|  |  | DOC | 1 | 0.000683 | 0.006827 | 0.13 | 0.73 |
|  |  | DIC x DOC | 1 | 0.00859 | 0.00858 | 1.63 | 0.238 |
|  |  | Aquaria | 8 | 0.0423 | 0.00529 | 1.77 | 0.180 |
|  |  | Residual | 12 | 0.035803 | 0.00298 |  |  |
|  |  | Total | 23 | 0.087464 |  |  |  |
|  | *H. opuntia* | DIC | 1 | 0.00375 | 0.00375 | 1.19 | 0.307 |
|  |  | DOC | 1 | 0.00336 | 0.0036 | 1.07 | 0.332 |
|  |  | DIC x DOC | 1 | 0.00251 | 0.0198375 | 6.31 | **0.036*** |
|  |  | Aquaria | 8 | 0.02516 | 0.0014 | 1.54 | 0.241 |
|  |  | Residual | 12 | 0.024491 | 0.00204 |  |  |
|  |  | Total | 23 | 0.00766 |  |  |  |
